# Nanomolar pyrophosphate detection and nucleus staining in living cells with simple terpyridine–Zn(II) complexes

**DOI:** 10.1038/srep26477

**Published:** 2016-05-20

**Authors:** Duobin Chao, Shitan Ni

**Affiliations:** 1 School of Petroleum and Chemical Engineering, Dalian University of Technology, Panjin, Liaoning 124221, P. R. China

## Abstract

Great efforts have been made to develop fluorescent probes for pyrophosphate (PPi) detection. Nucleus staining with fluorescence microscopy has been also widely investigated. But fluorescent probes for PPi detection with high sensitivity in water medium and nucleus staining with low–cost non–precious metal complexes in living cells are still challenging. Herein, we report simple terpyridine–Zn(II) complexes for selective nanomolar PPi detection over ATP and ADP in water based on aggregation induced emission (AIE) and intramolecular charge transfer (ICT). In addition, these terpyridine–Zn(II) complexes were successfully employed for nucleus staining in living cells. These results demonstrated simply obtained terpyridine–Zn(II) complexes are powerful tool for PPi detection and the development of PPi–related studies.

Anions play important roles in a variety of biological and environmental processes. Pyrophosphate (PPi) anion has received a lot of attention due to its considerable role in cellular metabolic processes and several diseases such as ATP hydrolysis and calcium pyrophosphate dihydrate crystal deposition disease^1^,^2^,^3^,^4^,^5^. Fluorescent probes for the detection of PPi are promising and widely studied, accompanied with the development of fluorescence microscopy in the past decades^6^,^7^,^8^,^9^,^10^,^11^,^12^,^13^,^14^. However, fluorescent probes with low limit of detection such as nanomolar level under physiological environments are attractive and still challenging mainly due to its high solvation energy of PPi in water. To overcome this obstacle, aggregation–induced emission (AIE) can be an efficient strategy^15^,^16^,^17^. AIE has been utilized as a promising strategy to develop fluorescent probes for the detection of many ions and biomolecules due to its simplicity and sensitivity in water medium^18^,^19^,^20^,^21^. However, rare probes for PPi are based on the formation of nanoaggregates utilizing AIE strategy. It is known that PPi has strong coordination affinity with metal ions such as Zn^2+^ and ensembles of Zn^2+^ are mostly employed for PPi detection with receptors including bis(2–pyridylmethyl) amine (DPA) and terpyridine derivatives^22^,^23^,^24^,^25^. PPi can coordinate with ensembles of Zn^2+^ and then induce variation of emission. Inspired by these results, we proposed a strategy for PPi detection by combination of its coordination affinity with Zn^2+^ and AIE as illustrated in Fig. 1.

Besides, fluorescence imaging cellular nucleus with luminescent dyes has also been widely utilized in biological research^26^,^27^,^28^,^29^. There are mainly two classical nuclear stains including organic dyes and transition metal complexes. Organic dyes such as 4′, 6–diamidino–2–phenylindole (DAPI) and Hoechst are well–known and commercially available nuclear stains, which are excited with UV light suffering from some drawbacks of photobleaching, selfquenching and autofluorescence of biological tissues. Transition metal complexes such as Ru(II) and Ir(III) complexes are also investigated for imaging nucleus, which usually display yellow or red emission with long lifetimes and can be used for time–resolved luminescence imaging^30^,^31^,^32^,^33^. However, these noble metal elements are rare, expensive and adventitious for many living organisms. Nuclear stains based on earth–abundant and non–precious metal elements are more attractive in view of widespread commercial applications. On the other hand, most of nuclear stains are based on the DNA stains^34^ Developing new strategies for imaging nucleus remains a significant challenge, for example, staining nucleus by imaging species besides DNA such as PPi in nucleus.

Recently, terpyridine derivatives and their Zn(II) complexes have emerged as one–photon and two–photon dyes used in bioimaging due to their rich photophysical properties^35^,^36^,^37^,^38^. Terpyridine derivatives can also be used for constructing many kinds of supramolecular assemblies with metal ions such as Zn^2+^, known as coordination–driven self–assembly^39^,^40^,^41^,^42^,^43^,^44^. These supramolecular assemblies have wide applications such as photocatalysts and sensors^45^,^46^,^47^. On the other hand, Zn(II) ion, as a “boring element” due to its inert redox and magnetic properties, is non–precious and widely present in living organisms, whose compound can be good potential candidate for imaging nucleus^48^. Keeping the above ideas in mind, in this paper, simple terpyridine–Zn(II) complexes were synthesized and employed for nanomolar PPi detection and nucleus staining.

## Results

### Fluorescence response to PPi using CZtpyZn

**Figure 2:**
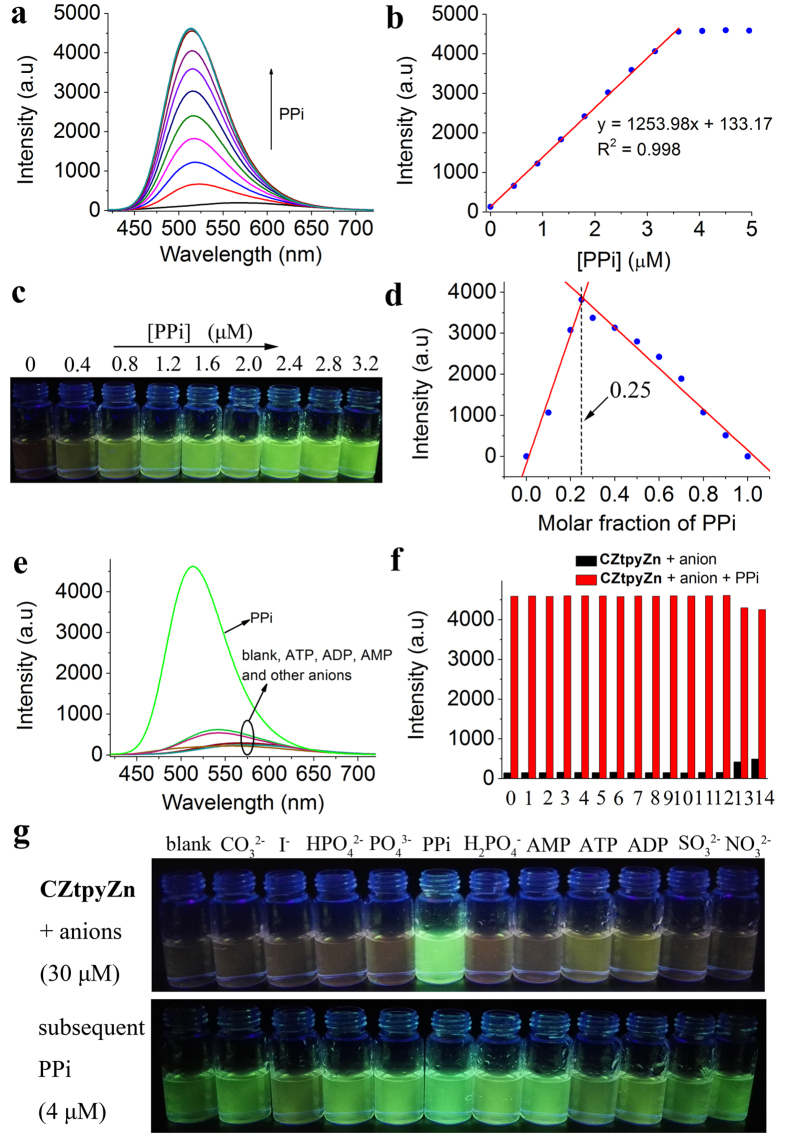
Detection of PPi with CZtpyZn. (**a**) Fluorescence spectra of **CZtpyZn** (10 μM) upon addition of PPi (0–5 μM) in HEPES buffer (pH = 7.4, 10 mM in H_2_O/DMSO, 7/3, v/v) when excited at 400 nm at room temperature. (**b**) Plot of fluorescence intensity at 515 nm versus concentration of PPi. (**c**) Photograph of **CZtpyZn** in the presence of PPi under a 365 nm UV lamp. (**d**) Job plot of binding study between PPi and **CZtpyZn.** The total concentration of PPi and **CZtpyZn** was controlled to be 10 μM. (**e**) Fluorescence spectra of **CZtpyZn** (10 μM) in the presence of anions (4 μM for PPi and 30 μM for other anions) when excited at 400 nm in HEPES buffer (pH = 7.4, 10 mM in H_2_O/DMSO, 7/3, v/v) at room temperature. (**f**) Fluorescence responses (515 nm) of **CZtpyZn** (10 μM) to various anions (30 μM) without PPi (black bar) and with PPi (4 μM, red bar). (namely 0–blank, 1–CO_3_^2−^, 2–SO_4_^2−^, 3–HSO_4_^−^, 4–Br^−^, 5–I^−^, 6–NO_3_^−^, 7–CH_3_COO^−^, 8–SO_3_^2−^, 9–H_2_PO_4_^−^, 10–HPO_4_^2−^, 11–PO_4_^3−^, 12–AMP, 13–ADP, 14–ATP) (**g**) Corresponding photographs under a 365 nm UV lamp.

To assess the possibility of **CZtpyZn** for the detection of PPi, its fluorescence response to PPi was first studied in HEPES buffer (pH = 7.4, 10 mM in H_2_O/DMSO, 7/3, v/v). **CZtpyZn** exhibited weak orange emission at 570 nm. However, upon addition of PPi dissolved in water, the emission showed a blue shift to 515 nm with green emission, accompanied with significant enhancement of intensity (Fig. 2). The green emission could be directly observed by naked eye even though the concentration of PPi was 0.4 μM (0.04 equivalent of **CZtpyZn**) (Fig. 2c). Good linear relationship was also observed between fluorescence intensity and concentration of PPi from 0.4 μM to 3.2 μM (Fig. 2b), suggesting potential application in the detection of PPi using **CZtpyZn**. Furthermore, the emission intensity was almost saturated when the equivalent of PPi was about 0.32 to **CZtpyZn**, indicating a binding stoichiometry of 1:3 (PPi: **CZtpyZn**). On the other hand, the Job plot verified this binding stoichiometry (Fig. 2d), in which the molar fraction of PPi was 0.25. The limit of detection (LOD) of PPi with **CZtpyZn** was found to be 5.37 nM (S/N = 3), indicating **CZtpyZn** is a highly sensitive probe for the detection of PPi.

Stimulated by the highly sensitive detection of PPi using **CZtpyZn**, we further studied its ability of detecting PPi in more complicated environment, especially in the presence of other competing nucleotide phosphates such as ATP, ADP and AMP. As illustrated in Fig. 2e, three equivalent (30 μM) of anions such as CO_3_^2−^, SO_4_^2−^, HSO_4_^−^, Br^−^, I^−^, NO_3_^−^, CH_3_COO^−^, SO_3_^2−^, H_2_PO_4_^−^, HPO_4_^2−^ and PO_4_^3−^ led to negligible changes for the emission of **CZtpyZn** (10 μM). Nucleotide phosphates including ATP, ADP and AMP also induced less effects on the emission compared with PPi. The competitive experiments showed that all anion solutions displayed green emission after subsequent addition of 0.4 equivalent (4 μM) PPi, even though weak yellow emission was observed in presence of ATP and ADP before addition of PPi (Fig. 2f,g), indicating **CZtpyZn** is a fluorescent probe for selective detection of PPi over ATP and ADP who usually interfere the detection of PPi. These results revealed that **CZtpyZn** is highly selective towards PPi.

### UV–vis absorption response to PPi using CZtpyZn

**Figure 3:**
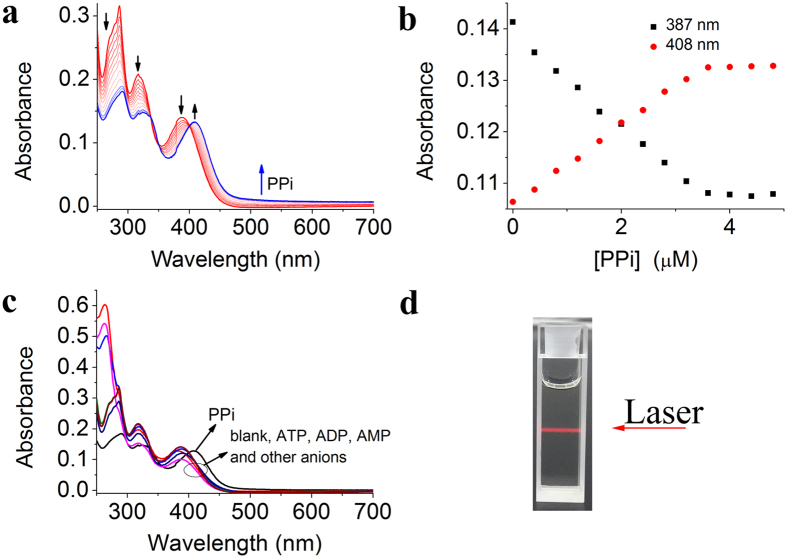
UV–vis absorption spectra of **CZtpyZn** in the presence of analytes (**a**) UV–vis absorption spectra of **CZtpyZn** (10 μM) upon addition of PPi (0–5 μM) in HEPES buffer (pH = 7.4, 10 mM in H_2_O/DMSO, 7/3, v/v). (**b**) Plot of absorbance at 387 nm and 408 nm of **CZtpyZn** versus concentration of PPi. (**c**) UV–vis absorption spectra of **CZtpyZn** (10 μM) in the presence of other anions (30 μM). (**d**) Photograph of a Tyndall effect in day light.

In order to gain more insights about the interaction between PPi and **CZtpyZn**, we next investigated the UV–vis absorption spectra of **CZtpyZn** in the presence of PPi. As shown in Fig. 3a, the UV–vis absorption spectrum of **CZtpyZn** showed three bands at 285 nm, 316 nm and 387 nm. However, the addition of increasing concentration of PPi resulted in dramatic change of UV–vis absorption spectra, in which the absorbance band at 387 nm gradually decreased and a new band at 408 nm appeared (Fig. 3b). A clear isosbestic point was observed at 400 nm, which indicated the formation of new species owing to the binding between PPi and **CZtpyZn**. It is noteworthy that a level–off tail above 500 nm was also observed. However, no similar level–off tail was observed for **CZtpyZn** in the presence of other anions and nucleotide phosphates (Fig. 3c). The appearance of a level–off tail is probably ascribed to light scattering effect of nanoaggregates^49^, suggesting the formed species might be nanoaggregates composed of supramolecular assembly of PPi and **CZtpyZn** in 1:3 binding mode. Furthermore, we observed a Tyndall effect when the solution of **CZtpyZn** in the presence of PPi was irradiated with a laser pointer (Fig. 3d), indicating the existence of nanoaggregates.

### Aggregation studies

**Figure 4:**
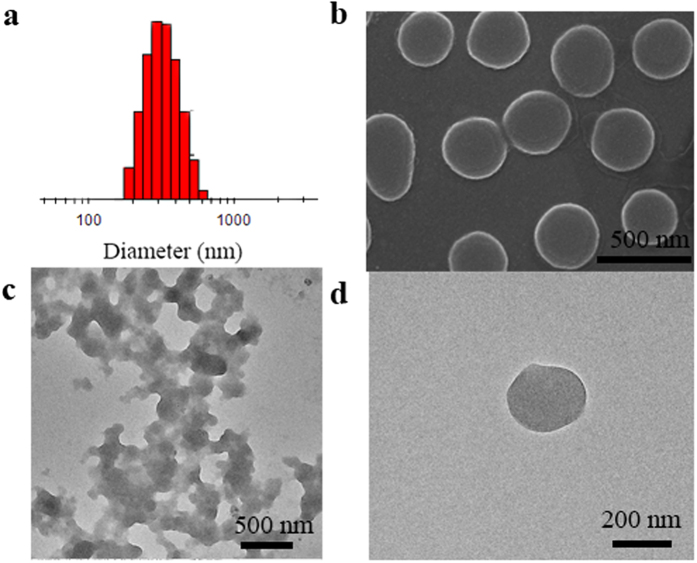
Confirmation of Nanoaggregates (**a**) DLS result of **CZtpyZn** (10 μM) after addition of PPi (4 μM) in HEPES buffer (pH = 7.4, 10 mM in H_2_O/DMSO, 7/3, v/v). (**b**) SEM image of **CZtpyZn** under the same condition. (**c**,**d**) TEM images of **CZtpyZn** under the same condition.

To confirm the formation of nanoaggregates, DLS, SEM and TEM experiments were further carried out. As shown in Fig. 4a, the average particle size was about 300 nm by DLS result for **CZtpyZn** in the presence of PPi. In addition, particles were also observed by SEM (Fig. 4b). However, TEM experiments suggest these particles tended to form nanoaggregates (Fig. 4c,d). Therefore, nanoaggregates were indeed formed during sensing process. To the best of our knowledge, this is the first example that demonstrated the formation of nanoaggregates when using Zn(II)–based ensembles for the detection of PPi.

### Nucleus staining with CZtpyZn

**Figure 5:**
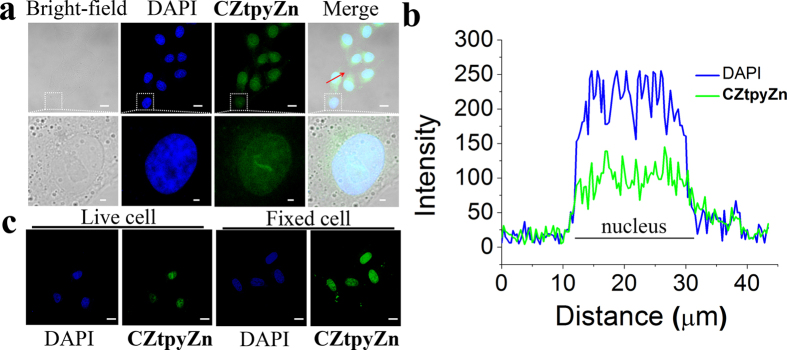
Nucleus staining with CZtpyZn. (**a**) The confocal fluorescence images of HeLa cells incubated with **CZtpyZn** (5 μM) for 30 min and then further incubated with DAPI (5 μg mL^−1^) for 10 min. Blue channel for DAPI excited at 405 nm and green channel for **CZtpyZn** excited at 488 nm. (**b**) Fluorescence profile of intensity across the red line in (**a**). (**c**) The confocal fluorescence images of living HeLa cells and fixed HeLa cells with **CZtpyZn**. Scale bar: 20 μm.

Considering that PPi are widely existing in biological fluids at micromole (μM) level, which is far beyond the LOD of **CZtpyZn**, we further employed **CZtpyZn** in cell imaging with HeLa cells. Before the cell imaging experiments, we evaluated the fluorescence response to PPi of **CZtpyZn** at different pH in water (Supplementary Fig. 1). We found that **CZtpyZn** are capable of detecting PPi at physiological pH (pH = 6–8) in water. Then, interestingly, as shown in Fig. 5a, **CZtpyZn** mainly lighted up the nucleus of HeLa cells, while very weak emission in the cytoplasm. Although the emission intensity of **CZtpyZn** was weaker than that of DAPI, **CZtpyZn** stained cells with same region as DAPI (Fig. 5b). Low cytotoxicity was also found by the MTT experiments (Supplementary Table 1). In addition, **CZtpyZn** can also be used for imaging fixed cells (Fig. 5c). The control experiment indicated green emission was mainly ascribe to PPi (Supplementary Fig. 2). Therefore, **CZtpyZn** can be utilized for nucleus staining.

### Cell imaging with AMtpyZn

**Figure 6:**
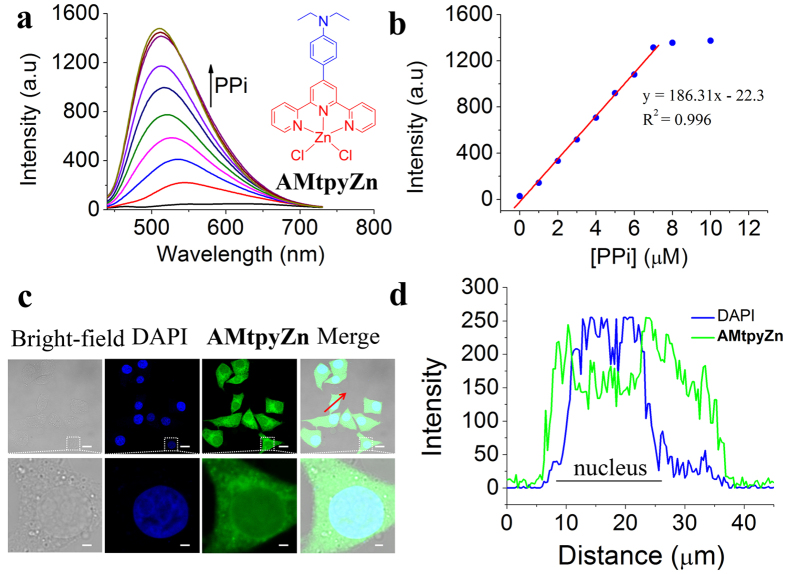
PPi detection and cell imaging with AMtpyZn. (**a**) Molecular structure of **AMtpyZn**. and Fluorescence spectra of **AMtpyZn** (20 μM) upon addition of PPi (0–10 μM) in HEPES buffer (pH = 7.4, 10 mM in H_2_O/DMSO, 99/1, v/v) when excited at 400 nm at room temperature. (**b**) Plot of fluorescence intensity of **AMtpyZn** at 520 nm versus concentration of PPi. (**c**) The confocal fluorescence images of HepG2 cells incubated with **AMtpyZn** (5 μM) for 30 min and then further incubated with DAPI (5 μg mL^−1^) for 10 min. Blue channel for DAPI excited at 405 nm and green channel for **AMtpyZn** excited at 488 nm. (**d**) Fluorescence profile of intensity across the red line in (**c**). Scale bar: 20 μm.

To manipulate the staining behavior of terpyridine–Zn(II) complexes, another new amino–modified terpyridine–Zn(II) complexes (**AMtpyZn**) was also facilely synthesized and developed for the detection of PPi in HEPES buffer (pH = 7.4, 10 mM in H_2_O/DMSO, 99/1, v/v) and imaging cells (Fig. 6). The solubility of **AMtpyZn** is better than that of **CZtpyZn** in water, so minimum amounts of DMSO was used. **AMtpyZn**, as a fluorescent probe for PPi, showed detection results similar to **CZtpyZn** such as turn–on fluorescence response to PPi (Fig. 6a), saturated fluorescence intensity when the equivalent of PPi was about 0.32 to **AMtpyZn** (Fig. 6b), a level–off tail in UV–vis absorption spectra (Supplementary Fig. 3), a Tyndall effect (Supplementary Fig. 4) and the suitable pH range (Supplementary Fig. 5), indicating that **CZtpyZn** and **AMtpyZn** bear the same mechanism for the detection of PPi such as formation of nanoaggregates. The limit of detection (LOD) of PPi with **AMtpyZn** was determined to be 9.72 nM (S/N = 3). We also employed **AMtpyZn** for cell imaging. However, **AMtpyZn** displayed different cell staining behavior from that of **CZtpyZn**, which lighted up a whole cell (Fig. 6c). As shown in Fig. 6d, DAPI mainly stained the nucleus, while **AMtpyZn** can stain both nucleus and cytoplasm, indicating the staining behavior of terpyridine–Zn(II) complexes can be manipulated by modifying their molecular structure.

## Discussion

Both **CZtpyZn** and **AMtpyZn** were facilely synthesized, starting from commercially available reagents (Supplementary Fig. 6). They are composed of earth–abundant, non–precious and biocompatible elements, suggesting their potential commercial applications. They both bear donor–acceptor (D–A) structure, in which carbazole and amino group are donors and terpyridine–Zn(II) part is the acceptor. Therefore, intramolecular charge transfer (ICT) effect was observed. In fact, terpyridine–Zn(II) complexes bearing D–A structure displayed different emission in solvents with various polarities (Supplementary Fig. 7), especially weak emission in aqueous solution due to strong polarity of water. However, when terpyridine–Zn(II) complexes aggregated to form nanoaggregates, the quenching effect caused by polar solvent such as water can be inhibited efficiently. The supramolecular assemblies composed of PPi and terpyridine–Zn(II) complexes are easily aggregated due to conjugated structure and low solubility. Hence, we observed strong emission known as AIE when nanoaggregates formed in the presence of PPi in water medium. Besides, the strong negative charge of PPi can weaken the ICT effect of terpyridine–Zn(II) complexes and contribute to emission change of terpyridine–Zn(II) complexes. As a result, the detection of PPi using terpyridine–Zn(II) complexes is ascribed to AIE and ICT owing to their coordination with PPi. We believe these results provide a promising strategy for anion detection such as PPi in water.

As mentioned, **CZtpyZn** mainly stained nucleus while **AMtpyZn** mapping a whole cell. Previous reports have illustrated that terpyridine derivatives ligands and their Zn(II) complexes bearing two terpyridine ligands were not capable of staining nucleus but only cytoplasm^37^,^38^. They are not able to coordinate with PPi or other species in nucleus. Therefore, **CZtpyZn** and **AMtpyZn** bearing one terpyridine ligand and liable leaving group (Cl^−^ anions) are capable of entering into cellular nucleus probably because of their coordination with phosphates in nucleus, especially PPi anions. This suggested that PPi anions not only induced strong emission of **CZtpyZn** and **AMtpyZn** but also PPi anions were helpful for their uptake and entry into cellular nucleus. The different staining behavior is ascribed to the structure difference between carbazole group and amino group. The carbazole–based compounds are widely studied owing to its donor ability, good planarity, rich photophysical properties and excellent biocompatibility^50^,^51^,^52^. In addition, carbazole derivatives are easily modified and obtained from commercial methods. As a consequence, low–cost carbazole–modified terpyridine–Zn(II) complexes have potential applications for developing commercially available nuclear stains in cell imaging field. More importantly, as is known to us, DNA stains for nucleus staining are popular and significantly investigated^34^. Herein, we provide an available strategy for cellular nucleus staining by imaging endogenous species such as PPi in living cells.

In summary, we successfully developed simple terpyridine–Zn(II) complexes for nanomolar PPi detection in water medium based on AIE and ICT. Nanoaggregates were confirmed in the detection processes. The carbazole–modified terpyridine–Zn(II) complex has a LOD of 5.37 nM and the amino–modified terpyridine–Zn(II) complex has a LOD of 9.72 nM. These results demonstrated that anion–induced aggregation emission is a powerful strategy for anion detection in water. Furthermore, taking advantage of the high sensitivity towards PPi, we employed them in cell imaging and found that the carbazole–modified terpyridine–Zn(II) complex mainly lighted up the nucleus and the amino–modified terpyridine–Zn(II) complex stained a whole cell. We believe the simply obtained and low–cost terpyridine–Zn(II) complexes have great potential applications in PPi–related studies such as cell imaging and diagnosis.

## Methods

### Materials and measurements

All materials were purchased from commercial suppliers and used as received. Cell lines were obtained from the lab of Prof. Zhigang Xie in Changchun Institute of Applied Chemistry Chinese Academy of Sciences. ^1^H NMR spectra were carried out using a Bruker Avance 500 MHz instrument with tetramethysilane (TMS) as an internal standard. HRMS spectra were recorded using a Thermofisher Q–Exactive instrument. Emission spectra were measured by Lengguang Tech. Instruments (F97PRO) with Xe lamp as the light source. UV–vis absorption spectra were obtained on a PerkinElmer Lambda 950 spectrophotometer. The scanning electron microscope (SEM) images were carried out on a Hitachi S–4800 scanning electron microscope. TEM image was recorded on JEM–2100 at 200 kV. The solid state quantum yield was measured using a calibrated integrating sphere on a Quantaurus–QY spectrophotometer. Particle size was determined with Malvern Nano ZS. Confocal Laser Scanning Microscope experiments for cell imaging were carried out with ZEISS LSM 510 according to the literature procedure^53^.

#### Synthesis of CZtpyZn

The synthetic route is shown in Supplementary Fig. 5. Solution of ZnCl_2_ (18.7 mg, 0.14 mmol) in CH_3_OH (5 mL) was added into a solution of the carbazole–modified terpyridine ligand^54^ (59 mg, 0.14 mmol) in CH_2_Cl_2_ (8 mL). The solution was stirred at 40 °C for 3 h. Then the precipitate was filtered, washed with water, methanol, and diethyl ether. The pure product was obtained after dried under vacuum. Yield: 47.0 mg, 60%. ^1^H NMR (500 MHz, DMSO–d6), δ (ppm): 9.21 (d, 3 H, J = 8.0 Hz), 9.03 (d, 2 H, J = 8.0 Hz), 8.86 (d, 2 H, J = 9.0 Hz), 8.47 (d, 1 H, J = 8.0 Hz), 8.37 (m, 3 H), 7.89 (m, 3 H), 7.73 (d, 1 H, J = 8.0 Hz), 7.56 (t, 1 H, J = 7.5 Hz), 7.35 (t, 1 H, J = 7.5 Hz), 4.58 (m, 2 H), 1.40 (m, 3 H). ESI–HRMS (m/z): found 525.0824 for [M–Cl]^+^ (calcd. for C_29_H_22_ClN_4_Zn 525.0824).

#### Synthesis of AMtpyZn

AMtpyZn was prepared using the same procedure as CZtpyZn, but the carbazole–modified terpyridine ligand was replaced by the amino–modified terpyridine ligand^55^. Yield: 36 mg, 50%. ^1^H NMR (500 MHz, DMSO–d_6_), δ (ppm): 8.97 (m, 4 H), 8.84 (d, 2 H, J = 9.0 Hz), 8.34 (m, 2 H), 8.21 (d, 2 H, J = 9.0 Hz), 7.87 (m, 2 H), 6.89 (d, 2 H, J = 9.0 Hz), 3.49 (m, 4 H), 1.18 (m, 6 H). ESI–HRMS (m/z): found 479.0980 for [M–Cl]^+^ (calcd. for C_25_H_24_ClN_4_Zn 479.0981).

## Additional Information

**How to cite this article**: Chao, D. and Ni, S. Nanomolar pyrophosphate detection and nucleus staining in living cells with simple terpyridine–Zn(II) complexes. *Sci. Rep.* **6**, 26477; doi: 10.1038/srep26477 (2016).

## Supplementary Material

Supplementary Information

## Figures and Tables

**Figure 1:**
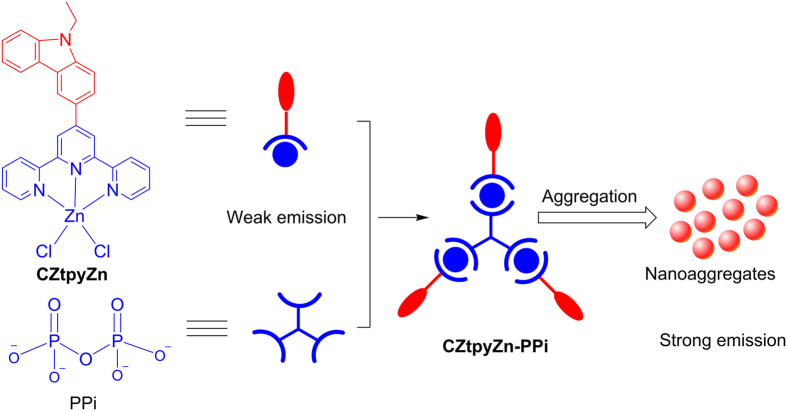
Proposed strategy for the detection of PPi in water medium. Weakly emissive terpyridine–Zn(II) complex (**CZtpyZn**) coordinates with PPi to form supramolecular assembly (**CZtpyZn–PPi**) and subsequently aggregates with strong emission.
