# Supplementary material for: Nanomolar pyrophosphate detection and nucleus staining in living cells with simple terpyridine–Zn(II) complexes
Source: Sci Rep. 2016 May 20;6:26477. doi: 10.1038/srep26477 (PMC4873787; doi:10.1038/srep26477)
Supplement: Supplementary Information [file srep26477-s1.pdf]

Supplementary Information

Nanomolar Pyrophosphate Detection and Nucleus Staining in living cells with Simple Terpyridine-Zn(II) Complexes

Cell imaging

For Imaging of Living Cells. The cells were grown with DMEM (containing 10% fetal bovine serum and antibiotics) for 24 h. The cells were then washed with HEPES buffer (pH = 7.4) and incubated with 5 μM **CZtpyZn** or **AMtpyZn** in DMEM for 30 min at 37 ^{o}C. After imaging, the cells further incubated with DAPI for another 10 min for imaging. Green channel was used for imaging with **CZtpyZn** or **AMtpyZn** and blue channel was used for DAPI.

For Imaging of Fixed Cells. The cells were detached from the culture and were fixed with 4% paraformaldehyde at room temperature for 20 min. After washing with HEPES buffer (pH = 7.4), the fixed cells were incubated with 5 μM **CZtpyZn** in DMEM for 30 min at 37 ^{o}C, and then further stained with DAPI for another 10 min. After washing with HEPES buffer, the coverslips were separated from the chamber, and the cells were mounted with 10% glycerol and sealed with nail varnish on a glass substrate.

Cell viability assays

The cytotoxicity of **CZtpyZn** and **AMtpyZn** was assessed with a 3–(4,5–dimethyl–2–thiazolyl)–2,5–diphenyl–2–H–tetrazolium bromide (MTT) assays towards HeLa cells and A549 cells. The cells were seeded in 96–well plates at about 10000 cells per well in 100 μL DMEM, and incubated at 37 ^{o}C in 5% CO_2 atmosphere for 24 h. After removing culture medium, CxNBDP NPs diluted in DMEM (100 μL) were added to cell wells with various concentrations of 1, 5, 10, 15, 20 and 25 μM. The cells were incubated for another 24 h. After the incubation, the culture medium was removed and DMEM (200 μL) was added into cell wells. Then 20 μL of 5 mg/mL MTT assays were added to cell wells and cells were incubated for another 4 h, followed by removal of the culture medium containing MTT and addition of 150 μL of DMSO to each well to dissolve the formazan crystals formed. Finally, the plates were shaken for 5 min. The absorbance of the solution was measured on a Bio–Rad 680 microplate reader at 490 nm. Cell viability (%) was calculated based on the following equation: $(A_{sample}/A_{control}) \times 100$ %, where A_{sample} and $A_{control}$ denote as absorbencies of the sample well and control well, respectively.

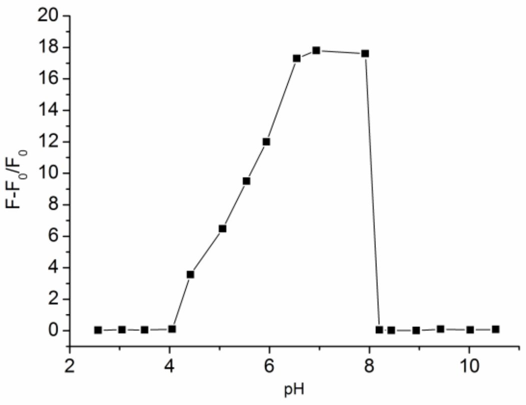

Supplementary Figure 1. Fluorescence intensity of **CZtpyZn** (10 μM) in the presence of PPi (4 μM) in HEPES buffer (pH = 7.4, 10 mM in H_2O/DMSO, 99/1, v/v) at room temperature. (λ_{ex} = 400 nm, λ_{em} = 515 nm)

Supplementary Table 1. Cytotoxicity of **CZtpyZn** and **AMtpyZn** by MTT assay.

Concentration	**CZtpyZn**	**AMtpyZn**
(μM)	HeLa	HepG2
0	100	100
5	92.0±2.5	91.5±2.0
10	90.2±1.9	89.2±1.7
15	88.5±3.2	87.6±2.3
20	83.4±2.0	83.5±1.5
25	80.6±1.7	80.0±3.1

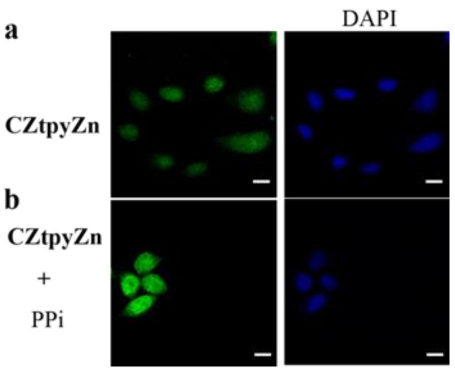

Supplementary Figure 2. (a) The confocal fluorescence images of HeLa cells incubated with **CZtpyZn** (5 μM) for 30 min and then further incubated with DAPI (5 μg mL^{-1}) for 10 min. (b) The confocal fluorescence images of HeLa cells incubated with **CZtpyZn** (5 μM) for 30 min and subsequently incubated with PPi (5 μM) for 10 min. Then further incubated with DAPI (5 μg mL^{-1}) for 10 min. Scale bar: 20 μm.

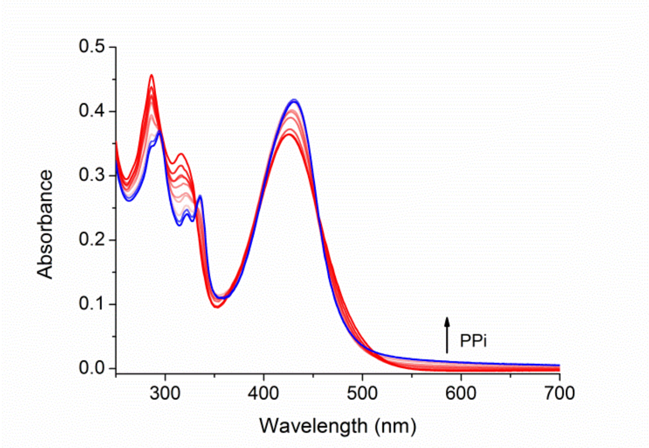

Supplementary Figure 3. UV–vis absorption spectra of **AMtpyZn** (20 μM) upon addition of PPi (0–10 μM) in HEPES buffer (pH = 7.4, 10 mM in H_2O/DMSO, 99/1, v/v).

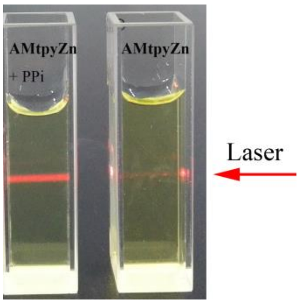

Supplementary Figure 4. Photograph of Tyndall effect of **AMtpyZn** (20 μM) in the presence of PPi (8 μM) in HEPES buffer (pH = 7.4, 10 mM in H_2O/DMSO, 99/1, v/v).

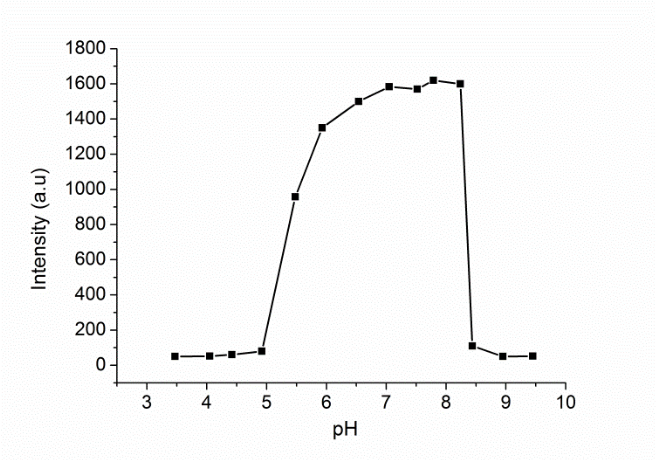

Supplementary Figure 5. Fluorescence intensity of **AMtpyZn** (20 μM) in the presence of PPi (8 μM) in HEPES buffer (pH = 7.4, 10 mM in H_2O/DMSO, 99/1, v/v) at room temperature. (λ_{ex} = 400 nm, λ_{em} = 520 nm)

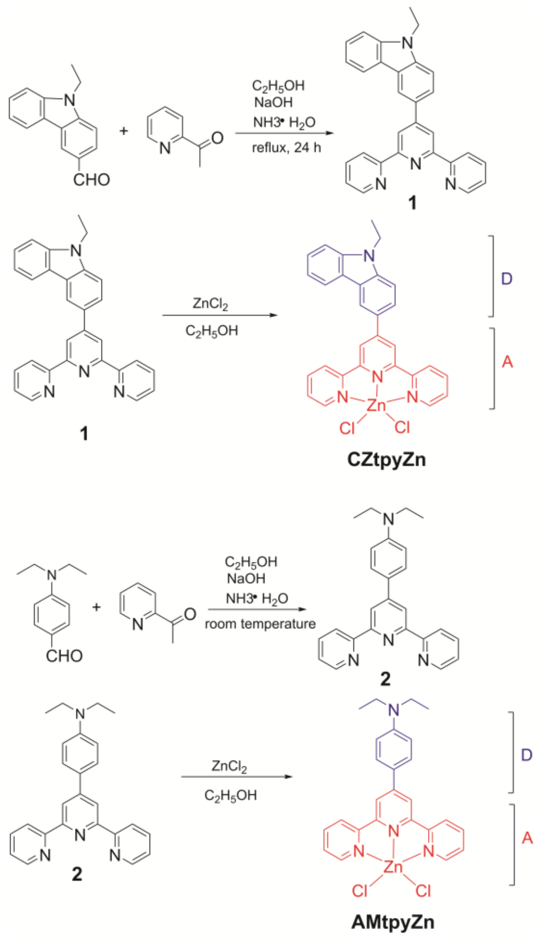

Supplementary Figure 6. Synthetic routes of **CZtpyZn** and **AMtpyZn**.

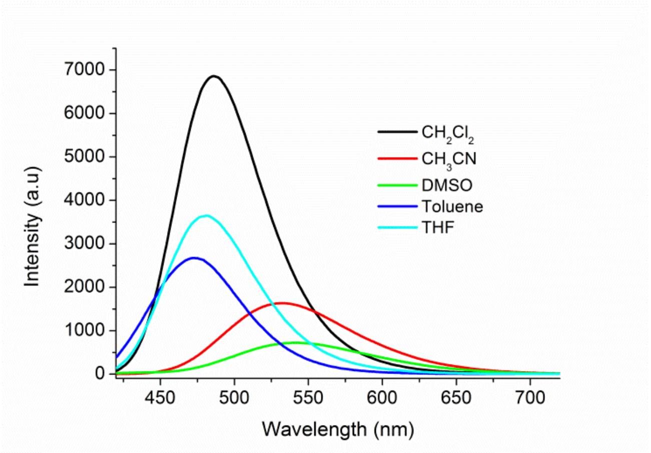

Supplementary Figure 7. Fluorescence emission spectra of **CZtpyZn** (10 μM) in different solvents.

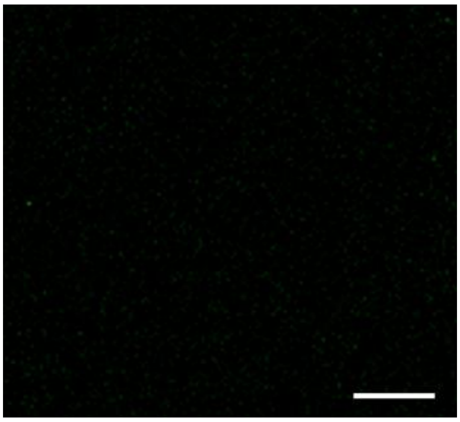

Supplementary Figure 8. The confocal fluorescence image of CZtpyZn (5 μM) in presence of PPi (3 μM) in aqueous solution in HEPES buffer (pH = 7.4, 10 mM in H_2O/DMSO, 7/3, v/v) with green channel. Scale bar: 20 μm.

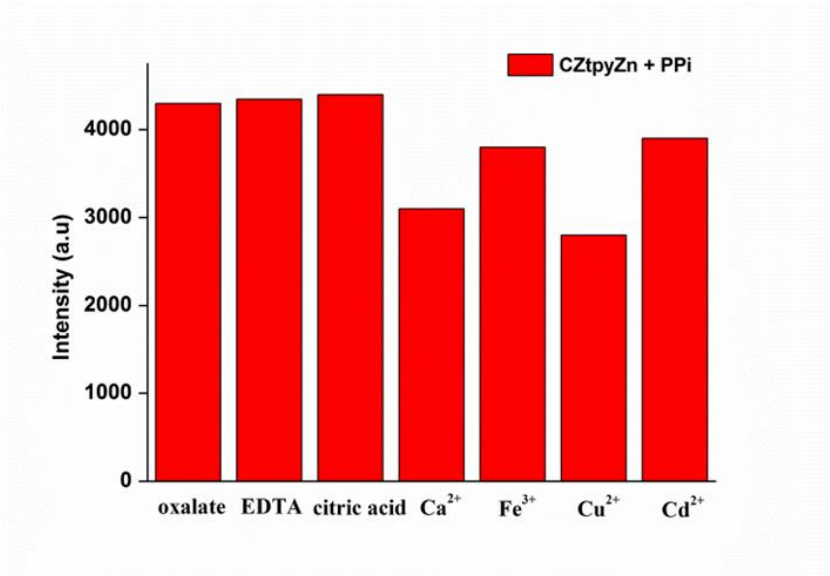

Supplementary Figure 9. Fluorescence responses (515 nm) of **CZtpyZn** (10 μM) in presence of PPi (4 μM)and other species (30 μM) including oxalate, EDTA, citric acid, Ca^{2+}, Fe^{3+}, Cu^{2+} and Cd^{2+} in aqueous solution in HEPES buffer (pH = 7.4, 10 mM in H_2O/DMSO, 7/3, v/v).

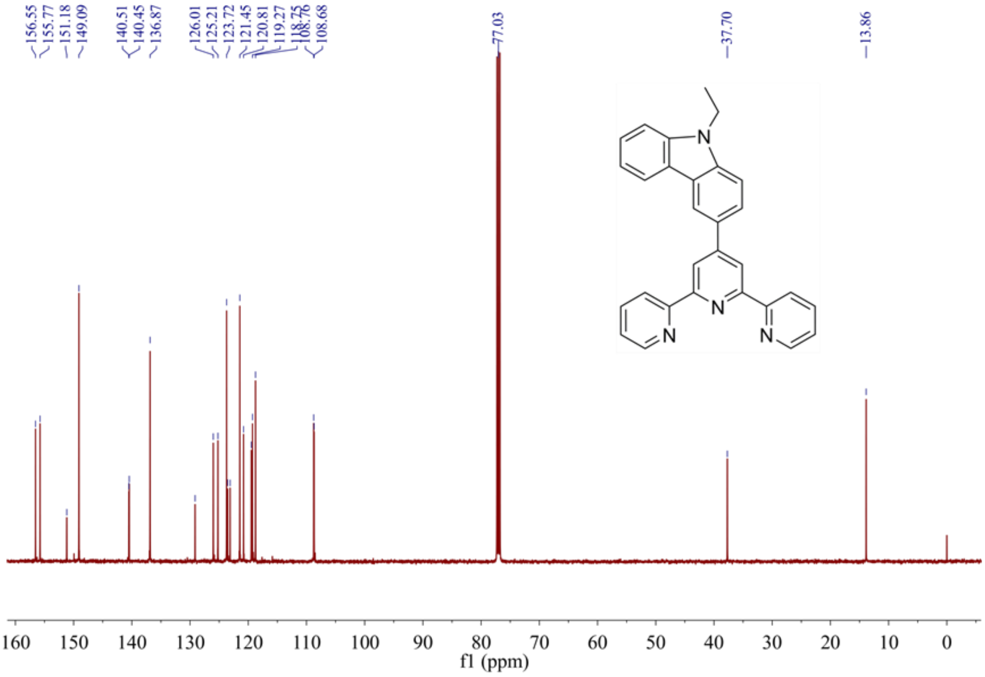

Supplementary Figure 10. ^{13}C NMR spectrum of **1** in $CDCl_3$ (125 MHz).

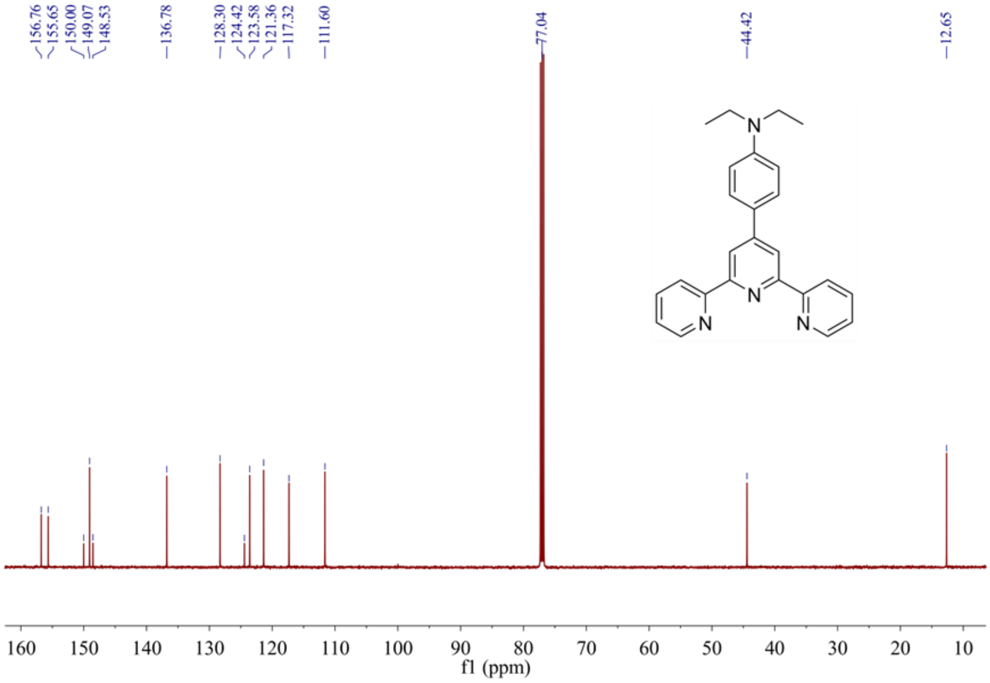

Supplementary Figure 11. ^{13}C NMR spectrum of **2** in $CDCl_3$ (125 MHz).

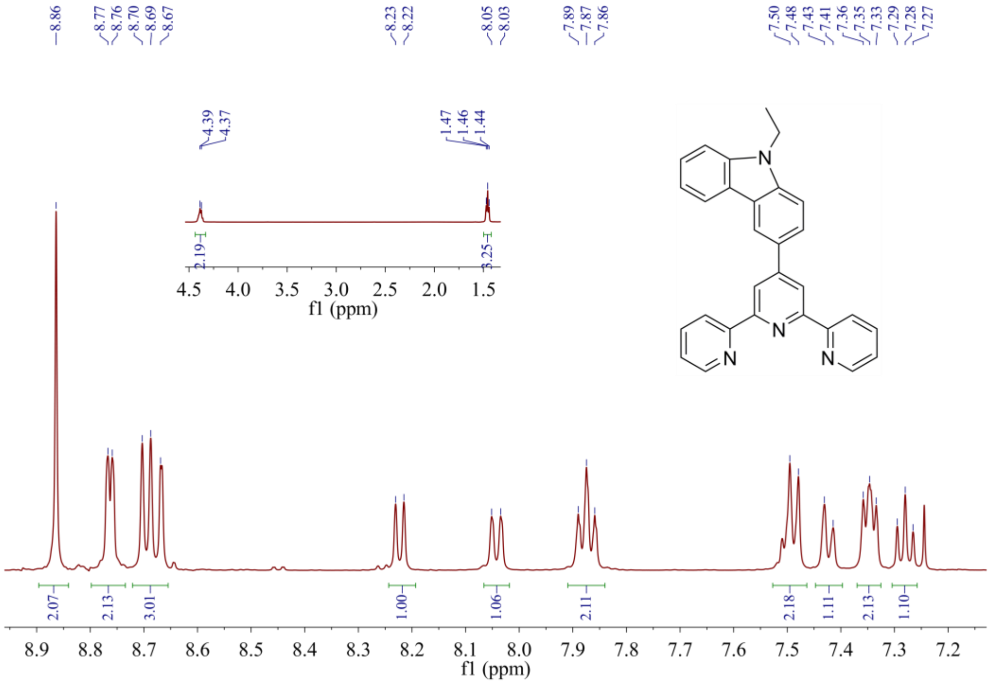

Supplementary Figure 12. ^{1}H NMR spectrum of **1** in $CDCl_3$ (500 MHz).

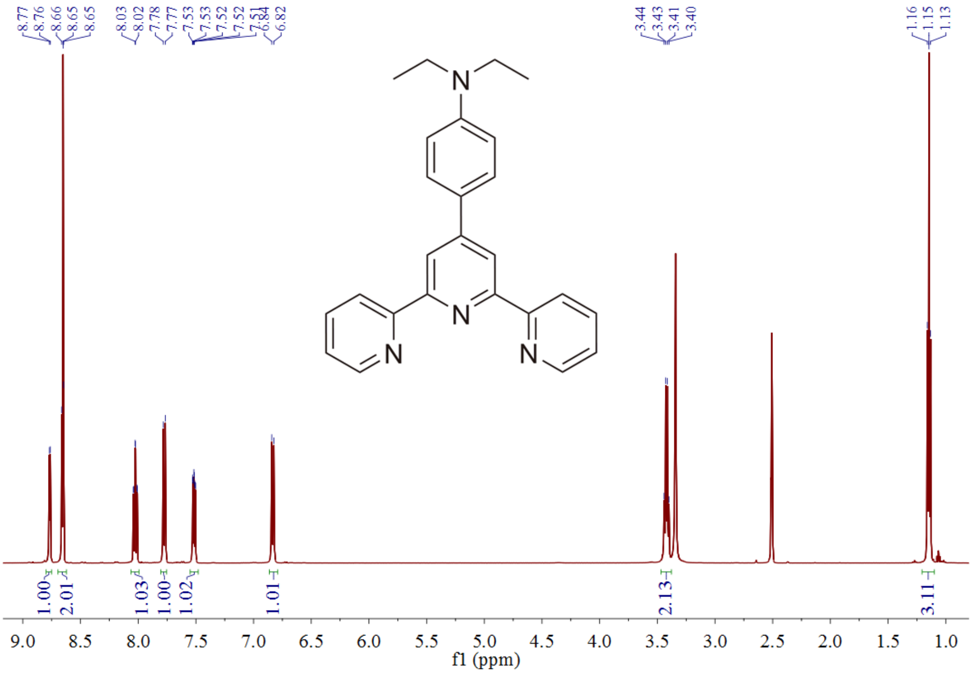

Supplementary Figure 13. ^{1}H NMR spectrum of **2** in DMSO–d_6 (500 MHz).

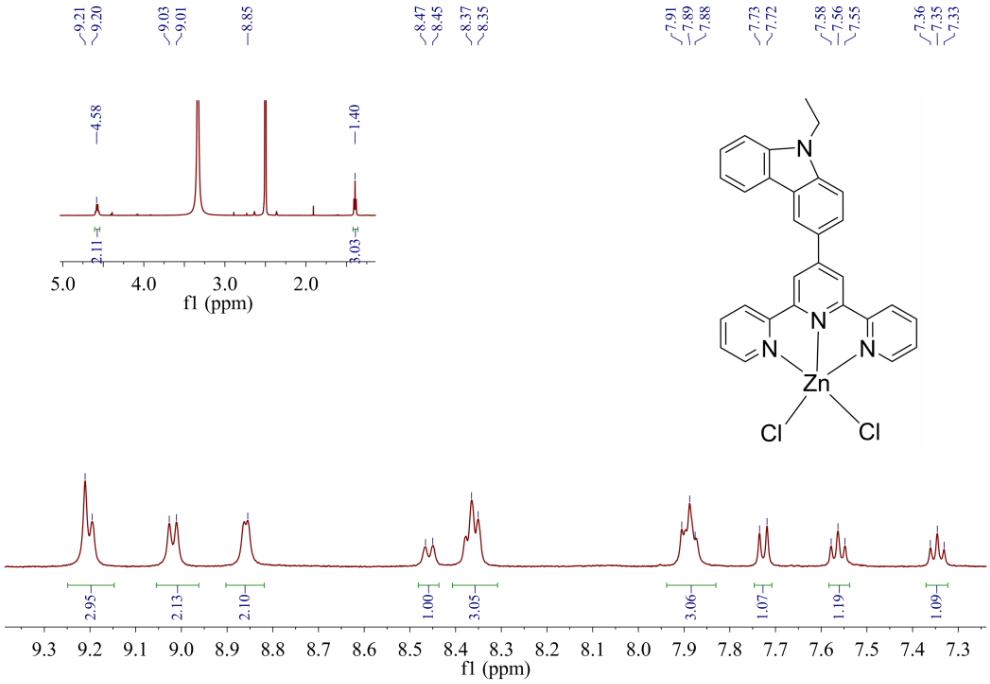

Supplementary Figure 14. ^{1}H NMR spectrum of **AMtpyZn** in DMSO–d_6 (500 MHz).

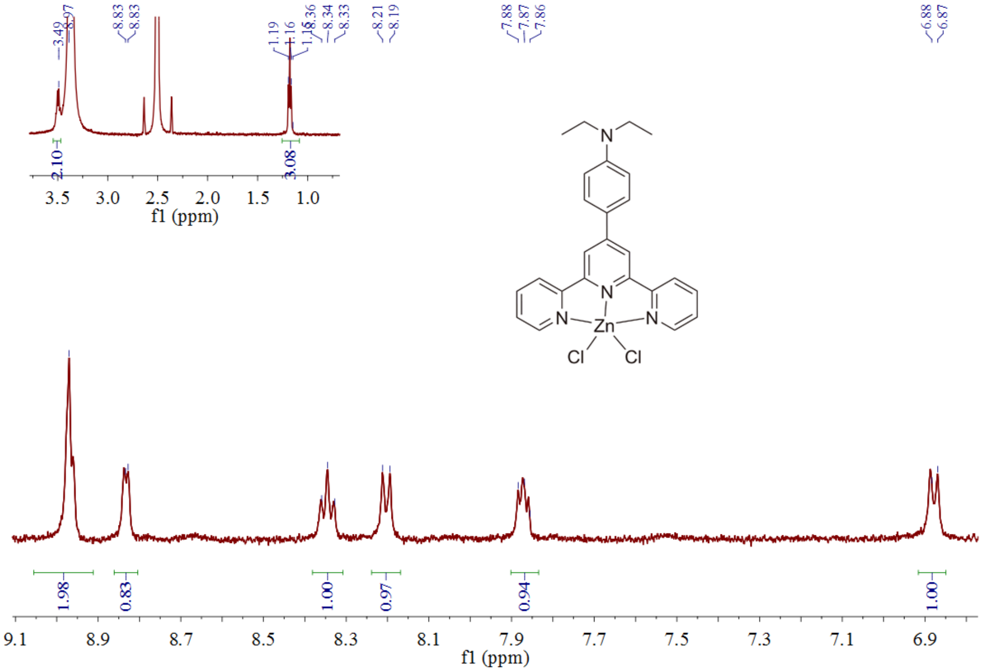

Supplementary Figure 15. ^{1}H NMR spectrum of **AMtpyZn** in DMSO–d_6 (500 MHz).

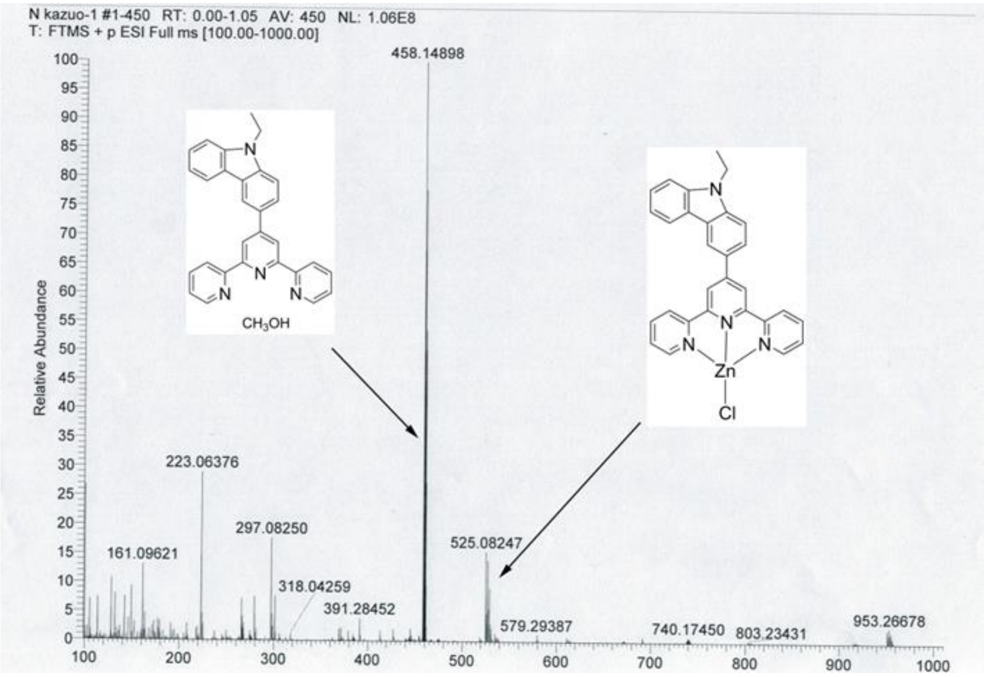

Supplementary Figure 16. ESI-MS spectrum of **CZtpyZn** in CH_3OH.

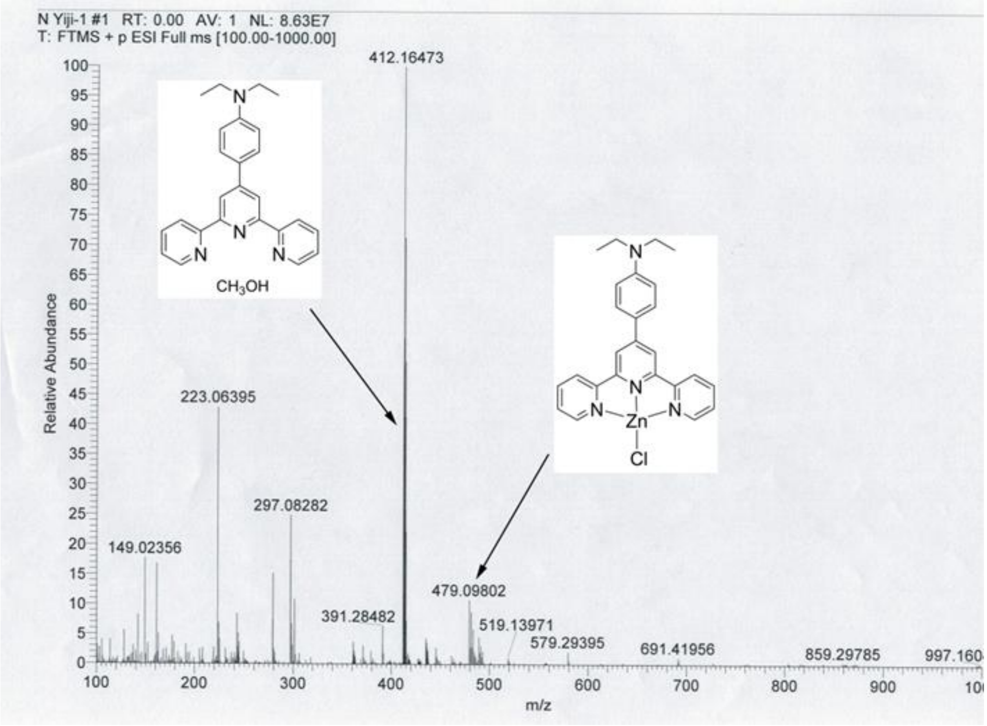

Supplementary Figure 17. ESI-MS spectrum of **AMtpyZn** in CH_3OH.
